# Research on the optimization of public health emergency response systems in higher education institutions

**DOI:** 10.3389/fpubh.2026.1761868

**Published:** 2026-02-18

**Authors:** Ping Wang, Xueli Yao, Jie Yu, Yan Chen, Fangfang Huang, Xinjuan Pan, Yingjian Zhang

**Affiliations:** 1 School of Basic Medicine and Forensic Medicine, Henan University of Science and Technology, Luoyang, China; 2 Department of Gastroenterology, The First Affiliated Hospital of Henan University of Science and Technology, Luoyang, China

**Keywords:** early warning mechanism, education, emergency management system, public, public health, public health emergencies in universities

## Abstract

**Objectives:**

In response to the frequent occurrence of public health emergencies in universities and the inadequacies in prevention and control systems, this study aims to establish a scientific and effective early warning mechanism and emergency management system to enhance universities' response capabilities and ensure the health and safety of teachers and students.

**Methods:**

A combination of literature review and empirical questionnaire surveys was adopted. Domestic and international literature on emergency management in universities was systematically analyzed to identify existing issues. Based on questionnaire data from 556 students at a university in Henan, their awareness levels, psychological states, and information acquisition channels regarding public health emergencies were assessed. Practical cases were also incorporated to validate effectiveness.

**Results:**

While current early warning mechanisms and emergency management systems have seen significant improvements, gaps remain. This study summarizes recent issues and actionable measures in a review format, supplemented by practical measures from a specific university, providing theoretical support for addressing public health emergencies in universities. This further safeguards the physical and mental well-being of teachers and students, ensuring social harmony and stability.

## Introduction

In recent years, with the increase of the population base, people's living standards have grown rapidly, and the incidence of public health emergencies has also increased rapidly. Due to the occurrence of emergencies has caused relatively serious damage to the people's physical and mental health and to the country's social and economic development. People are also gradually aware of the dangers of “public health emergencies,” and the degree of attention has gradually deepened. At the same time, the number of students in colleges and universities has continued to rise, and due to the complexity of student origins, dense populations, and fixed university venues, schools have become key protection sites for public health emergencies (1). As a “nest” for cultivating outstanding talents, colleges and universities should integrate harmony and tranquility to provide students with a good learning environment. However, the characteristics of fixed places and high-density gathering points make it easy to become the best vector for the spread of public health events. In order to avoid incidents, an early warning mechanism and an emergency management system should be established and improved in a timely manner.

In this study, a comprehensive key university in central China with science, engineering and medicine as the main focus and multidisciplinary coordinated development was selected as the research object. The university covers an area of 4,600 acres, covering 11 university disciplines, with 98 undergraduate majors, and has more than 44,000 full-time undergraduates, postgraduates, international students, and 24,000 continuing education students. The school has more than 3,300 faculty members and about 60,000 students. The students cover 31 provincial-level administrative regions across the country, and have the typical characteristics of dense population and strong mobility. Based on the empirical research carried out by the university (including a questionnaire survey of 556 students), the sample representativeness is mainly reflected in the following aspects, including: (1) wide geographical coverage: the students of the university come from a wide range of regions and multiple provinces; (2) Distributed disciplines: including medical and non-medical majors, covering 8 university disciplines such as arts, science, engineering, agriculture, and medicine; (3) Rich experience in management practice: experience in handling emergencies.

## Methods

### Literature review method

Through systematic search of authoritative databases at home and abroad (such as CNKI, PubMed, Web of Science, etc.), policy documents, academic papers and case reports related to public health emergencies in universities in the past 10 years were collected. ref Selection criteria include:

- Topic scope: Focusing on the early warning mechanism, emergency management system and prevention and control strategy of public health emergencies in universities.
- Time frame: Priority will be given to the research results from 2013 to 2023 to ensure timeliness.
- Literature Type: Covers theoretical research, empirical analysis and policy evaluation.

This paper analyzes the content of the included literature, and sorts out several key issues in Italy and Spain: (1) the institutional differences and practical experience of the stress management system of universities at home and abroad; (2) the advantages and disadvantages of existing early warning technologies; and (3) the effective performance of psychological intervention measures. (4)The student background is diverse: the sample encompasses students from three different schools, various grade levels, and distinct majors, thereby reflecting the differences in cognition, behavior, and needs among different student groups.

### Questionnaires

A total of 600 medical students and non-medical students in the first to third grades of three colleges and universities in Henan Province were selected by stratified random sampling, and the ratio of medical majors and non-medical majors was 1:1. The selection criteria of the study subjects are as follows: (1) age ≥18 years old; (2) College degree or above; (3) Be able to complete the questionnaire independently in a voluntarily informed manner.

According to the relevant literature (2), the questionnaire was designed by itself and distributed to medical and non-medical students by means of online questionnaires, and students could fill in the questionnaire by scanning the QR code or clicking on the link. Based on the background of public health emergencies, the questionnaire evaluates the handling of emergencies by college students and colleges and universities facing public health emergencies from multiple perspectives, such as their basic knowledge and cognition of public health emergencies, students' psychological state of emergencies, and school emergency response. Invalid questionnaires are eliminated, valid questionnaires are numbered, and entered into the computer for calculation. Elimination criteria for invalid questionnaires: (1) blank questionnaires; (2) The answer rate is less than 80% of the questionnaire.

### Statistical processing methods

SPSS 25.0 software and SPSSPRO were used for statistical analysis, and the chi-square test statistical method was used, and *P* < 0.05 was used to indicate statistical significance. In terms of the analysis of school psychological intervention and students' access to information, a multi-response analysis method was adopted, and a detailed analysis was carried out based on the response rate and penetration rate.

## Result

### Public health emergencies in colleges and universities

#### Concepts related to public health emergencies in domestic universities

For the concept of public health emergencies in colleges and universities, please refer to the Regulations on Emergency Response to Public Health Emergencies promulgated in China in 2003: Sudden public incidents in institutions of higher education refer to unforeseen emergency events that occur without prior warning, take place on campus or are associated with members of the institution, and exert severe disruptive impacts on the personal and property safety of the institution, its faculty and students, as well as the operational order of teaching, research and administrative work. Internationally, in the International Health Regulations adopted in 2005, a public health emergency is defined as an unusual event which, through the international spread of disease, poses a public health risk to other countries and may require a coordinated international response. According to the Emergency Plan for Public Health Emergencies in the Education System, public health emergencies are generally classified into four levels: general, relatively large, major, and particularly serious, according to the severity of public health emergencies. Corresponding to emergency response levels I to IV respectively, and classified by category, they can be divided into five major types of events: natural disaster events, public health emergencies, sudden accidents, social security emergencies, and network information security incidents.

Public health emergencies in colleges and universities are difficult to predict before they occur, have a great impact on all aspects of the university, and even threaten social stability, and more than half of the public health emergencies occur in the university, so the scientific response to the emergency is a challenge that colleges and universities must face, and it is also an inescapable and arduous task (3)^.^ Emergencies are sudden, global, unconventional, publicness and dangerous (4).

The results of the survey data (Tables 1, 2) show that 92.4% of college students were able to obtain information about public health emergencies earlier, mainly through the Internet (90.5%) and official publicity channels (88.7%), indicating that the modern information dissemination system plays a key role in the early warning of events.

**Table 1:** Students' general information and the time when they were notified of emergencies.

**project**	**Medical students**	**Non-medical students**	**Proportion/%**	***X2***	***P***
Male/Female degree	51/224	116/165	30/70	34.187	0.000
Specialist and below	29	40	12.4		
Undergraduate	207	190	71.4	4.017	0.134
Master's degree or above know the time	39	51	16.2		
The first time	255	259	92.4		
Not the first time	13	13	4.7	0.216	0.897
Unclear	7	9	2.9		

The time of learning is expressed as the time when the student learns the news after the emergency occurs.

**Table 2:** Survey of students' access to information.

**Channels for obtaining information**	***N***	**Response ratio/%**	**Popularizing rate/%**	***X^2^***	***P***
News reports on television or radio	513	21.600	92.300	249.899	< 0.0001^***^
Publicity by government departments, schools and communities	493	20.700	88.700		
Mobile phone browsing	503	21.200	90.500		
Informing family members and friends	420	17.700	75.500		
Newspapers and periodicals	270	11.400	48.600		
Else	177	7.400	31.800		
Aggregate	2376	100.000	427.338		

^***^ Represent the significance levels of 10%, respectively.

#### Public health emergencies in foreign universities

As early as the 60s of the 20th century, the emergency management of Western colleges and universities has gradually been paid attention to, but China's understanding of emergency management started very late, nearly 50 years later than the West, and the management of this aspect was in 2003 after SARS, in view of the experience of teaching, in 2005 the Ministry of Education promulgated and implemented the “Education System Emergency Plan for Public Emergencies”. By the 70s of the 20th century, the “Practical Guide to Campus Crisis Response” was more influential abroad. Countries such as the United States, Japan, France and the United Kingdom have long been paying attention to the emergency management of colleges and universities, and the highest level of public crisis management in the United States is Federal Emergency Management Agency (FEMA), which has made campus crisis management more organized. Some scholars have found that after the outbreak of emergencies, American university libraries actively responded to the policy, established a variety of emergency response organizations, united with libraries to carry out online services, formulated emergency response mechanisms, and carried out online service teaching (5)^.^ In terms of prevention, the United States has established a “GSERP” to recruit graduate-level public health students to provide state and local health departments with the ability to respond quickly, as well as to provide students with training in applying public health experience and specific activities (6) to respond quickly in the face of emergencies. Expanding access to datasets by the research community in the aftermath of a co-health event (7). Japan attaches great importance to campus safety and has improved crisis management in colleges and universities on the basis of law. As a disaster-prone country with frequent earthquakes and volcanoes, Japan has introduced a number of basic laws that can respond to disasters in order to respond quickly to emergencies. In addition, Japan has established emergency management departments in universities (8). French universities have established relatively comprehensive systems for campus medical services and campus police. The campus police, under government jurisdiction and vested with judicial authority, are primarily responsible for campus patrols, safeguarding the safety of faculty, students, and public property, and preventing emergencies, with high efficiency and good results (9). The United Kingdom has established specialized departments for early warning and response to public emergencies, and the British government will set up temporary bodies such as the Emergency Cabinet Committee to address public emergencies (10).

### Early warning mechanism for public health emergencies in colleges

#### Status of early warning mechanisms

In the literal sense, the early warning mechanism can be interpreted as an early warning system. The outbreak of SARS has exposed many problems in the early warning mechanism of public health emergencies in China's universities. With governments at all levels and universities attaching importance to the establishment of public health early warning mechanisms for emergencies and summarizing the lessons and lessons of previous emergencies, the early warning mechanism has tended to be perfected. Universities and relevant government departments have paid corresponding attention to the management of the early warning mechanism of public health emergencies in colleges and universities. However, there are still problems such as independent management departments, unsmooth communication channels, and incomplete national laws and regulations (11). Some scholars have pointed out that the specific responsibilities of China's early warning mechanism handling system have not yet been clarified, and there are many detection problems, and there are slow and complex problems in the systematic detection of public health emergencies (12). At present, public health emergencies include many data, such as health data, collection site situation information, medical information, and social data, and it is very difficult to manage human resources (13), and it is also a major problem to organize these complex multi-source data for the formation of automatic early warning of public health crises (14).

#### Establish early warning mechanisms, existing measures

Regarding the establishment of an early warning mechanism for public health emergencies in universities, some scholars proposed to use relevant technologies to make early warnings of public health emergencies in colleges and universities in view of the problems existing in the early warning mechanism. According to a foreign article, the construction of a sensor to integrate and analyze various data in the public area, including the situation and real-time health data, and the use of situational awareness to establish a new public health crisis early warning system, in order to carry out targeted epidemic prevention and control, the advantages of this technology are to reduce the risk of data management and reduce the cost of responding to public health crises, and have high research and application value (13).

With the development of the times, many scholars have designed and researched a variety of technical methods to improve the early warning mechanism in view of the shortcomings arising from the occurrence of emergencies. A scholar proposed the use of data warehouse technology to carry out specific applications in the early warning and control of public health emergencies in universities (15). Data warehouse technology collects, summarizes, and analyzes information to achieve information management, so that the incidence of various diseases and infectious diseases in schools can be monitored, and a platform for early warning and control can be formed (16)^.^ Transform the collected data into usable safety warning information to optimize and supplement the established school security management (17). Transform on the basis of the original old architecture, run new Internet of Things technology, and cooperate with GIS technology to quickly respond and select incident assistance addresses; Through load balancing technology, the Internet of Things (IoT) can be optimized to minimize unnecessary system development expenditures and system development time, so as to solve medical and educational problems in public health emergencies in a targeted manner (12).

#### A university's response to public health emergencies is a threefold improvement dimension

In view of the above status quo and measures, a university has held many meetings to study and evaluate the emergency situation, conveyed the spirit of the “Notice on Urgently Carrying out the ”Ten Strict Investigations“ of Epidemic Prevention and Control in Colleges and Universities in the Province” issued by the Provincial Headquarters Education System Work Task Force, further clarified the responsibilities of each work class of the school headquarters, and rearranged and redeployed the school's prevention and control work. The following improvement measures should be taken: first, optimize the command system, clarify the responsibilities of each special class, carry out the investigation of potential safety hazards in the whole school, and focus on strengthening the prevention and control of classrooms, dormitories, canteens and other dense places; The second is to strengthen normal management, implement prevention and control measures such as “wearing masks”, ventilation and sanitizing, and closed-loop management, and promote full coverage of vaccination; The third is to improve emergency preparedness, refine the prevention and control plan, strictly implement various epidemic prevention requirements, and build a solid campus safety line. Strengthen safety education for teachers and students in schools. Carry out safety education for teachers and students in fire protection, transportation, food and health and epidemic prevention, fire prevention, anti-theft, anti-fraud, etc., and effectively enhance the safety awareness of teachers, students and employees.

Based on the above methods, we investigated the cognitive judgment of medical and non-medical students on the relevant basic knowledge in public health emergencies (Table 3), and the results showed that the students of the university, regardless of whether they majored in medicine or not, showed a high level of awareness of the typical symptoms and main transmission routes of public health emergencies (the overall awareness rate was >90%, and the droplet contact transmission recognition rate was >96%).

**Table 3:** Symptoms and transmission modes of public health emergencies such as novel coronavirus pneumonia and tuberculosis among school students.

**Project**	**Student**	***N***	**Response ratio (%)**	**χ^2^**	***P***
**Symptoms**					
Fever and dry cough	Medical students	19	6.9	1.611	0.753
	Non-medical students	16	5.7		
	headcount	35	6.3		
Nasal congestion and runny nose	Medical students	3	1.1		
	Non-medical students	1	0.4		
	headcount	4	0.7		
Fatigue and condition may gradually worsen	Medical students	1	0.4		
	Non-medical students	1	0.4		
	headcount	2	0.3		
All of the above known	Medical students	252	91.6		
	Non-medical students	263	93.6		
	headcount	554	93.0		
**Modes of disease transmission**					
Blood-borne transmission	Medical students	6	2.2	2.492	0.458
	Non-medical students	2	0.7		
	headcount	8	1.4		
Vertical transmission	Medical students	4	1.5		
	Non-medical students	3	1.1		
	headcount	7	1.3		
Airborne and contact transmission	Medical students	264	96.0		
	Non-medical students	275	97.9		
	headcount	539	97.0		
Sexual transmission	Medical students	1	0.4		
	Non-medical students	1	0.4		
	headcount	2	0.4		

### Emergency management system for public health emergencies in colleges and universities

Emergency management in colleges and universities is to prevent and deal with crises in colleges and universities, and to manage the whole process of occurrence, with the aim of better solving and controlling crises after they occur, so as to ensure the personal safety of teachers and students and reduce social property losses to the greatest extent. Although the new crown pneumonia has brought harm to society, it has also made people continue to grow and progress from it. Emergency management in colleges and universities is a systematic work that runs through the whole process of crisis prevention, response and recovery, aiming to ensure the safety of teachers and students to the greatest extent and reduce losses. The prevention and control of the new crown epidemic has provided an important practical opportunity for the construction of the emergency response system of colleges and universities, and the following progress has been made in the past 3 years: (1) system optimization: most universities have established a three-level emergency response mechanism, and the decision-making efficiency has been significantly improved; (2) Ability improvement: The training coverage rate of management personnel has increased from 32 to 89%, and the frequency of drills has increased by three times. In order to improve the efficiency of emergency response, the questionnaire colleges and universities have established a 24-h joint prevention and control mechanism, and built a four-level linkage prevention and control system of “school-college-class-house” by clarifying the division of responsibilities at all levels, strengthening on-duty supervision, and implementing rewards and punishments. Strictly implement the requirements of people, objects and the environment, continuously improve emergency response capabilities through normalized supervision, and form a prevention and control pattern with full participation, coordination and efficiency.

However, there are still outstanding problems, mainly including: (1) 65% of the emergency procedures of colleges and universities have not been standardized and are not operable; (2) Students' emergency knowledge is lacking, and the correct cognition rate of non-medical students is less than 50%; (3) Poor coordination between departments, and the information sharing mechanism needs to be improved urgently; (4) Public health investment is insufficient, and the proportion of funding is generally less than 0.5%. These shortcomings need to be made through institutional innovation, capacity building, resource integration and other measures, to transform the experience of epidemic prevention and control into a long-term mechanism, and continue to improve the level of emergency management in universities.

In response to the above problems, we counted the emergency response of the university in the face of public health emergencies, and obtained the following results (Table 4). The results showed that the implementation of school entry and exit management was the best (*P* < 0.001), followed by material preparation (*P* < 0.001), and there was no significant difference in emergency plan cognition. To compensate for the limitations of the *P*-value, we added the effect size Cramer's V to quantify the strength of the association between categorical variables and determine the actual size of the difference. Through the above results, we need to focus on strengthening the publicity of emergency plans and give priority to ensuring the standardized implementation of school entry and exit management.

**Table 4:** Questionnaire survey on the emergency response to public health emergencies in schools.

**Project**	**Element**	***N***	**Response ratio (%)**	**χ^2^**	***P* value**	**Cramer's V**
Check at the school entrance and exit	Double-check	515	92.6	1256.33	< 0.001	0.89
	Just need the health code	17	3.1			
	Only measure body temperature	12	2.2			
Material preparation	No inspection	12	2.2	68.21	< 0.001	0.35
	Adequate	437	78.6			
	Inequacy	34	6.1			
status contingency plan	Unknown	85	15.3	5.67	0.129	0.12
	Yes	438	78.8			
	No	35	6.3			
	Unknown	83	15.0			

### Mental health education in colleges and universities

Song Xueqi talked about the management strategy of the emergency management system under the influence of the epidemic, and proposed to focus on students with psychological abnormalities to help them do psychological enlightenment (18). After an emergency, students hope that their daily lives can return to normal as soon as possible, and they are eager to be understood and supported by others, which provides the basis for crisis intervention (19). This study systematically evaluated the effectiveness of psychological interventions in schools after public health emergencies through questionnaire surveys (Table 5) and explored the emotional response characteristics of medical and non-medical students in the face of similar events (Table 6). Among the results, the prevalence rate of psychological questionnaire survey and counseling was the highest (84.5%), which was better than other measures, and the response rate of psychological questionnaire was the highest (31.7%). Psychology lectures/courses covered 81.3% of students, and the response rate (30.5%) complemented the class station (26.7%). A three-level psychological intervention system of “questionnaire screening, lecture intervention, and daily monitoring” has been formed. Under this system, students' psychological state formed a form of positive coping as the mainstream (90.5% of medical students and 97.2% of non-medical students). The proportion of anxiety and fear was significantly higher among medical students (6.2%) than that of non-medical students (2. 1%) (*P* = 0.006). The attitude of “nothing to do with oneself” only existed in the medical student group (1.5%), and there were significant professional differences in the distribution of psychological states. We conducted a risk assessment of the differences between medical and non-medical students, mainly including anxiety risk and negative attitude risk (none of their business + others), although medical students were more knowledgeable, they showed a higher risk of anxiety, which may be related to the following factors: (1) professional cognitive burden: medical knowledge may amplify risk perception; (2) occupational exposure anxiety: alertness to pathogens that are more sensitive; (3) Training pressure: the uncertainty caused by the interruption of clinical practice during the epidemic.

**Table 5:** Psychological interventions taken in schools.

**Psychotherapy**	***N***	**Responsivity/%**	**Popularizing rate/%**	**χ^2^**	***P***
Conduct a series of psychological education lectures and courses	452	30.500	81.300	160.251	< 0.0001^***^
Conduct psychological questionnaire surveys and provide timely and appropriate counseling	470	31.700	84.500		
Establish a class emotional support center and observe students' daily behaviors.	395	26.700	71.000		
Else	165	11.100	29.700		
Aggregate	1,482	100.000	266.547		

^***^Represents a 10% significance level, reflecting the results of the multiple response analysis. The total number of responses was 1482, with an average of 2.67 intervention measures per student.

**Table 6:** Statistical analysis results of the difference in psychological state between medical.

**Project**	**Factor**	**Student**		**Response ratio**		**Statistical magnitude**	
		**Medical students**	**Non-medical students**	**Medical students**	**Non-medical students**	***X** ^2^*	***P***
Psychological states	Psychological states	249	273	90.5	97.2	11.215	0.006
	Anxiety, fear	17	6	6.2	2.1		
	Not Interested	4	0	1.5	0		
	Else	5	2	1.8	0.7		
						**RR**	**95%CI**
Risk analysis	Anxiety risk	17	6	6.2	2.1	2.95	1.18-7.38
	Negative attitude risk	9	2	3.3	0.7	4.71	1.02-21.76

Negative attitude: It's not about you + anything else.

In public health emergencies, social mentality plays a crucial role in social risk early warning. Timely identification of specific manifestations of social mentality imbalance during public health emergencies, along with fostering and guiding social members to establish a healthy and positive social mentality, holds significant practical importance for effectively preventing and responding to public health emergencies, resolving public psychological crises, and maintaining social stability. Therefore, cultivating a positive social mentality, building a solid social psychological defense line, and thereby safeguarding national social security and stability, constitutes one of the important tasks for the Party and the country in effectively addressing public health emergencies in the new era. So in the event of an emergency, it is recommended that colleges and universities focus on the implementation of students‘ psychological and behavioral problems and preventive interventions, such as establishing a basic information database of students, paying close attention to students' psychological state, conducting regular psychological questionnaire surveys, screening out students with psychological abnormal tendencies, providing targeted and timely psychological counseling to students, popularizing common sense, reducing or even eliminating the negative emotions of relevant students, and avoiding risks as soon as possible. In addition, with the development of the Internet, the speed of message transmission has accelerated, which also means that a lot of untrue news can easily reach the ears of students, causing panic and other emotions among students.

The specific measures implemented by the questionnaire colleges and universities for mental health intervention are as follows: focusing on education and teaching, consulting services, preventive interventions, practical activities, and platform guarantees. Through these “five grasps”, the scientific and pertinent mental health education of college students can be improved. Establish a five-star classification standard for students' mental health status, screen key populations, and report the list to the Mental Health Center of the Student Affairs Department for specific consultation. During the consultation, the expert will evaluate the student's situation through the relevant student counselors and parents, and make an intervention opinion.

### Daily prevention of public health emergencies in colleges and universities

The main types of public health emergencies in colleges and universities include: student food poisoning; outbreaks and epidemics of infectious diseases (such as tuberculosis, AIDS, hepatitis B, diarrhea, etc.); occurrence of student accidental injuries, etc. At present, the incidence of AIDS is increasing year by year, colleges and universities are areas with a high incidence of AIDS, and in view of the fact that the current publicity in colleges and universities is not in-depth enough, it is necessary to carry out college education and publicity for students. The propaganda personnel present at the scene distributed pamphlets, played micro-videos, on-site explanations,

consultation questions and answers, and answered questionnaires, or displayed the cases of HIV-related infections, transmission mechanisms, and risky behaviors and scenarios to be prevented to the public in various forms such as micro-videos, games, paintings, handicrafts, and manuscripts, so as to popularize HIV/AIDS prevention knowledge among college students. Let teachers and students have more love for AIDS patients and less misinterpretation, so as to truly “join hands in epidemic prevention and fight AIDS and share health responsibilities”.

In terms of safety accidents, through the development of safety education courses, with recent safety accidents as warnings, we will thoroughly implement the spirit of the series of important instructions of General Secretary Jinping on safety production work, so that all units and departments can deeply learn the lessons of recent accidents and improve emergency response capabilities. The logistics department is used to carry out flood prevention drills to further enhance the awareness of flood prevention, improve the escape skills of accidents, and effectively strengthen teachers and students to master the escape methods when unexpected disasters come during the flood season, so as to effectively ensure the safety of the campus during the flood season.

In terms of food safety, in order to further implement food safety, the launching ceremony of the provincial food safety into the campus activity was carried out, and at the same time, the food safety emergency science popularization drill was carried out to improve the students' awareness of food safety prevention. In order to further optimize the ability and handling process to deal with emergencies, thoroughly implement the epidemic prevention and control work requirements of higher-level departments, further strengthen the epidemic prevention and control work of schools, and enhance the ability and mentality of teachers and students to deal with emergencies. In summary, public health emergencies in universities are complex in types and diverse in causes, posing challenges to emergency management.

## Discussion

In this study, we found that the perfection of the emergency management system of universities directly affects the response to public health emergencies. It runs through the entire process of emergency management, namely the four stages of crisis prevention, preparation, response, and recovery. Therefore, measures in the form of institutionalization, proceduralization, systematization, standardization, and theorization should be adopted to deal with it. Through the empowerment of technologies such as intelligent early warning systems and the innovation of multi-departmental collaborative mechanisms, universities can significantly improve the efficiency of emergency response. It is mentioned in the literature that government representatives and public health experts form a specific form of emergency response team as the network center, which is responsible for directing and coordinating the organization of the response network center, so as to improve the efficiency of emergency management (17). Furthermore, by implementing the theory of collaborative governance, the maximum utilization of resources can be achieved through cooperation and coordination among various governance entities in the emergency management of public emergencies in colleges and universities. Collaborative governance serves as a necessary means for innovating China's public management model under the new situation, thus making it more imperative to explore new approaches to public management in the context of the new era.

The results of this study are consistent with the relevant literature at home and abroad, and verify the core role of technology-driven management optimization in emergency management. Compared with western developed countries, the emergency management of universities in China started late, but through the practice of recent years (such as the prevention and control of the new crown epidemic), an emergency management system with Chinese characteristics has been gradually established. The “three-level psychological intervention system” and the “grid management model” proposed in this study are important supplements to the existing theories, especially in terms of student psychological support and departmental coordination.

Public health emergencies in colleges and universities have garnered widespread attention from all sectors of society, and doing a good job in managing such incidents is of great significance. Based on the empirical survey results of this study, sound emergency management can not only directly safeguard the physical and mental health of teachers and students—for instance, through standardized psychological interventions, the anxiety prevalence among non-medical students has been reduced to 2.1%—but also maintain campus harmony and tranquility, while laying a solid foundation for the harmonious development of society and the country. More importantly, colleges and universities are the core hubs for cultivating high-quality talents and advancing scientific and technological innovation in China. Looking at international practices, Japan's legal-based crisis management model and Italy's technological early warning systems both confirm that effective emergency management in colleges and universities is a strategic cornerstone for sustaining the normal order of teaching and scientific research, ensuring the integrity of the national talent and scientific research supply chains, and enhancing the risk resilience of higher education systems. This is not only crucial for safeguarding China's long-term competitiveness in talent cultivation and technological innovation but also bears on national overall security, which is aligned with the global consensus of prioritizing campus public health governance.

## Data Availability

The original contributions presented in the study are included in the article/supplementary material, further inquiries can be directed to the corresponding author.
