# Correction: Research on the optimization of public health emergency response systems in higher education institutions

**DOI:** 10.3389/fpubh.2026.1814278

**Published:** 2026-03-05

**Authors:** Ping Wang, Xueli Yao, Jie Yu, Yan Chen, Fangfang Huang, Xinjuan Pan, Yingjian Zhang

**Affiliations:** 1 School of Basic Medicine and Forensic Medicine, Henan University of Science and Technology, Luoyang, China; 2 Department of Gastroenterology, The First Affiliated Hospital of Henan University of Science and Technology, Luoyang, China

**Keywords:** early warning mechanism, education, emergency management system, public, public health, public health emergencies in universities

An incorrect Funding statement was provided. The correct funder is Research and Practice Project on Higher Education Teaching Reform in Henan Province (No. 2021SJGLX378). The correct funding statement reads:

“The author(s) declared that financial support was received for this work and/or its publication. This study was supported by Research and Practice Project on Higher Education Teaching Reform in Henan Province (No: 2021SJGLX378). The funders were responsible for providing financial support for the research design, data collection and statistical analysis, and did not participate in the writing of the manuscript and the decision to submit the article for publication.”

The original version of this article has been updated.

